# Acute pharmacological profile of 2C-B-Fly-NBOMe in male Wistar rats—pharmacokinetics, effects on behaviour and thermoregulation

**DOI:** 10.3389/fphar.2023.1120419

**Published:** 2023-03-09

**Authors:** Kateřina Syrová, Klára Šíchová, Hynek Danda, Eva Lhotková, Pascal Jorratt, Nikola Pinterová-Leca, Čestmír Vejmola, Lucie Olejníková-Ladislavová, Kateřina Hájková, Martin Kuchař, Jiří Horáček, Tomáš Páleníček

**Affiliations:** ^1^ Psychedelics Research Centre, National Institute of Mental Health, Prague, Czechia; ^2^ Third Faculty of Medicine, Charles University, Prague, Czechia; ^3^ Forensic Laboratory of Biologically Active Compounds, Department of Chemistry of Natural Compounds, University of Chemistry and Technology, Prague, Czechia

**Keywords:** 2C-B-Fly-NBOMe, NBOMe series, pharmacokinetics, prepulse inhibition, thermoregulation, new psychoactive substance

## Abstract

**Introduction:** *N*-2-methoxy-benzylated (“NBOMe”) analogues of phenethylamine are a group of new psychoactive substances (NPS) with reported strong psychedelic effects in sub-milligram doses linked to a number of severe intoxications, including fatal ones. In our present work, we provide a detailed investigation of pharmacokinetics and acute behavioural effects of 2C-B-Fly-NBOMe (2-(8-bromo-2,3,6,7-tetrahydrobenzo [1,2-b:4,5-b′]difuran-4-yl)-*N-*[(2-methoxybenzyl]ethan-1-amine), an analogue of popular psychedelic entactogen 2C-B (4-Bromo-2,5-dimethoxyphenethylamine).

**Methods:** All experiments were conducted on adult male Wistar rats. Pharmacokinetic parameters of 2C-B-Fly-NBOMe (1 mg/kg subcutaneously; s. c.) in blood serum and brain tissue were analysed over 24 h using liquid chromatography-mass spectrometry (LC/MS). For examination of behavioural parameters in open field test (OFT) and prepulse inhibition (PPI) of acoustic startle reaction (ASR), 2C-B-Fly-NBOMe (0.2, 1 and 5 mg/kg s. c.) was administered in two temporal onsets: 15 and 60 min after administration. Thermoregulatory changes were evaluated in individually and group-housed animals over 8 h following the highest dose used in behavioural experiments (5 mg/kg s. c.).

**Results:** Peak drug concentrations were detected 30 and 60 min after the drug application in serum (28 ng/ml) and brain tissue (171 ng/g), respectively. The parental compound was still present in the brain 8 h after administration. Locomotor activity was dose-dependently reduced by the drug in both temporal testing onsets. ASR was also strongly disrupted in both temporal onsets, drug’s effect on PPI was weaker. 2C-B-Fly-NBOMe did not cause any significant thermoregulatory changes.

**Discussion:** Our results suggest that 2C-B-Fly-NBOMe penetrates animal brain tissue in a relatively slow manner, induces significant inhibitory effects on motor performance, and attenuates sensorimotor gating. Its overall profile is similar to closely related analogue 2C-B and other NBOMe substances.

## 1 Introduction

New psychoactive substances (NPS) have become a persistent problem worldwide. According to the European Monitoring Centre for Drugs and Drug Addiction, it now consists of approximately 880 substances with 52 NPS first reported in Europe by the end of 2021 (EMCDDA, 2022). Clandestine laboratories produce legal, cheap, and easily accessible analogues (often called “bath salts’’, “legal highs” or “research chemicals”), which mimic the effects of established illicit drugs, although they are often more potent compared to parental drugs.

Currently, *N*-2-methoxybenzyl-substituted (NBOMe) phenethylamines are one of the most frequently appearing NPS on the illicit drugs market. They were initially developed for the research on 5-HT_2A_ receptor-mediated functions (Elz et al., 2002), but soon after they entered the black market. Data from drug-related online forums, such as PsychonautWiki, Erowid or Bluelight, show threshold dose for psychoactive effects as low as 50 μg, recreational doses usually range between 200 and 1,000 μg. They are mostly administered buccally or sublingually *via* blotting papers; although nasal sprays, pills, insufflation of powder or application of suppositories are also reported to some extent (Lawn et al., 2014). Onset and duration of psychoactive effects strongly depend on the route of administration, but onset is generally fast (15 min after sublingual intake), and the effects can last for up to several hours (8–10 h after sublingual intake). Recreational users on Erowid commonly describe NBOMes as mimicking combined effects of 3,4-methylenedioxymethamphetamine (MDMA) and lysergic acid diethylamide (LSD), with intense visual hallucinations, euphoria, deep empathy, intensive perception of art and music, as well as stimulatory effects with increased heart rate and body temperature. However, their intake already caused severe intoxications including fatal ones, caused either by unintentional ingestion of NBOMe (Suzuki et al., 2014), often marketed as “legal LSD” or milder 2C drugs, or by critical variations of drug content and various impurities on blotter papers (Poklis et al., 2015; Duffau et al., 2016). Highly intoxicated individuals can suffer from anxiety, confusion, panic attacks and paranoia, they can also manifest aggressive and bizarre behaviour. In terms of adverse somatic effects, intoxications with NBOMes can cause seizures, kidney failure, toxic leukoencephalopathy, or show clinical features associated with serotonergic toxicity, such as tachycardia, hypertension, breathing difficulties, convulsions, sweating and overheating, rhabdomyolysis, acute multiorgan failure, heart arrhythmia, fibrillation, and cardiac arrest (Hill et al., 2013; Poklis et al., 2014; Stellpflug et al., 2014; Humston et al., 2017; Richeval et al., 2017). Most frequent fatal poisonings involve 25I-NBOMe, 25B-NBOMe and 25C-NBOMe (Andreasen et al., 2015; Kueppers and Cooke, 2015; Nikolaou et al., 2015; Shanks et al., 2015; Al-Imam, 2018).

It is generally acknowledged that the hallucinogenic properties of drugs are mostly exerted through the activation of cortical 5-HT_2A_ receptors and subsequent glutamate release (Halberstadt, 2015; Schmitz et al., 2022; Wojtas et al., 2022). The addition of *N*-2-methoxybenzyl moiety to 2C drugs massively increases their binding affinity to 2A and 2C serotonin receptor subtypes, and moreover, binding to adrenergic α_1_, histaminergic H_1_, dopaminergic D_1_-_3_ receptors and monoamine transporters is also increased. However, affinity and potency of NBOMe analogues seem to be low at 1A and 2B serotonin receptor subtypes (Rickli et al., 2015; Eshleman et al., 2018).

**FIGURE 1:**
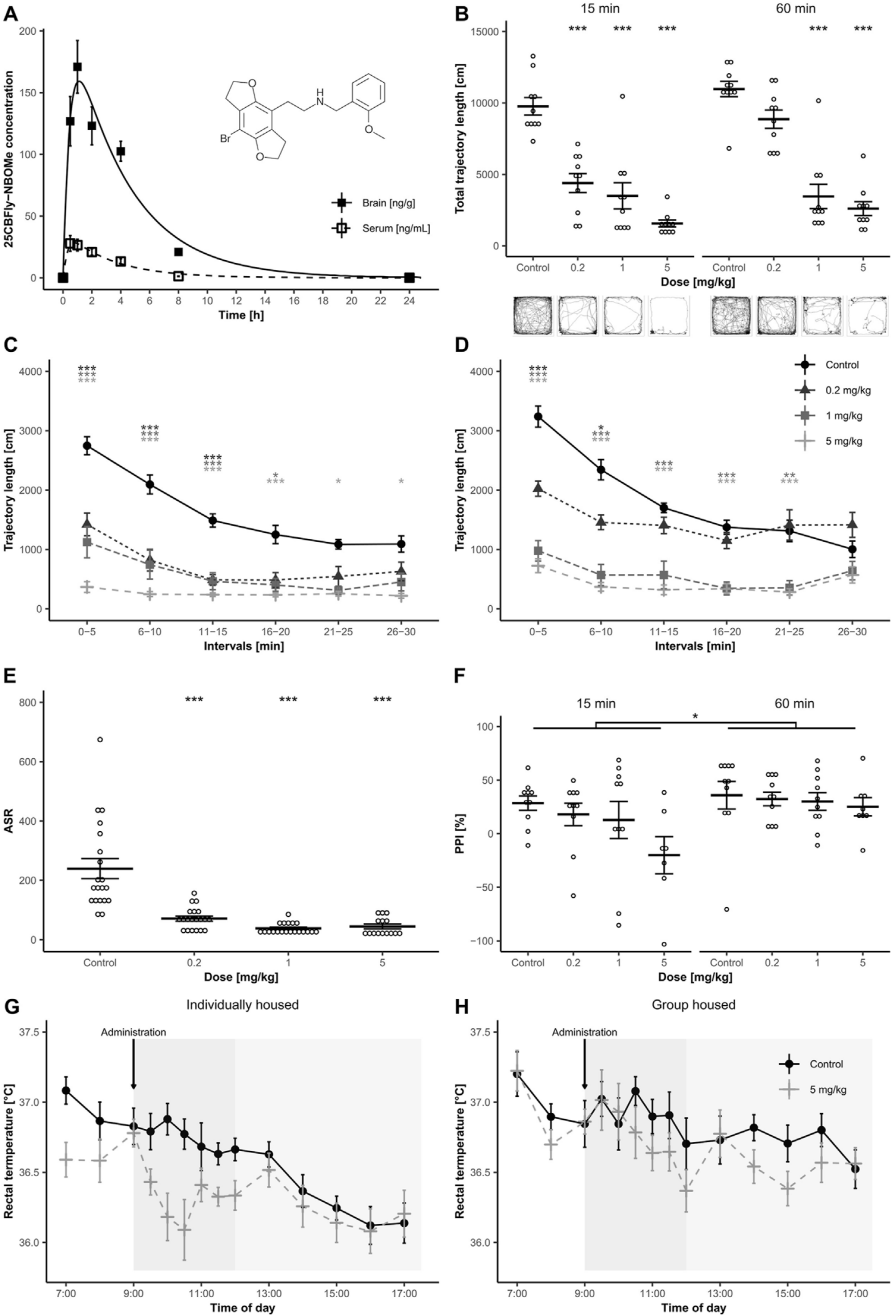
**(A)** Structure of 2C-B-Fly-NBOMe and graph of mean 2C-B-Fly-NBOMe concentration (1 mg/kg s. c.) in serum and brain over 24 h (n = 8 per time point). Error bars display ±1 standard error of the mean (SEM). **(B)** Total locomotion measured 15 and 60 min after 2C-B-Fly-NBOMe (0.2, 1 and 5 mg/kg s. c.) or ctrl administration (n = 10). Error bars display ±1 SEM, asterisks indicate significant differences from the ctrl group (*p < 0.05, **p < 0.01, ***p < 0.001). Picture inserts below demonstrate trajectory patterns induced by the drug treatment. **(C,D)**: Means of trajectory length within 5 min blocks 15 min **(C)** and 60 min **(D)** after administration of 2C-B-Fly-NBOMe (0.2, 1 and 5 mg/kg s. c.) or ctrl. Error bars display ±1 SEM, asterisks indicate significant differences from ctrl (*p < 0.05, **p < 0.01, ***p < 0.001). **(E)** Mean effect of 2C-B-Fly-NBOMe (0.2, 1 and 5 mg/kg s. c.) on ASR (n = 7–10). Error bars display ±1 SEM, asterisks indicate significant differences from ctrl (*p < 0.05, **p < 0.01, ***p < 0.001). **(F)** Comparison of mean PPI of ASR values of 2C-B-Fly-NBOMe (0.2, 1 and 5 mg/kg s. c.) and ctrl in 15 min **(A)** and 60 min **(B)** temporal onset (n = 7–10). Error bars display ±1 SEM, asterisks indicate significant differences from the ctrl (*p < 0.05, **p < 0.01, ***p < 0.001). **(G,H)**: The effect of 2C-B-Fly-NBOMe (5 mg/kg s. c.) on mean rectal temperature (°C) of individually housed animals **(G)** and 5 animals **(H)** per cage (n = 10). 2C-B-Fly-NBOMe or ctrl were administered at 09:00 h. Error bars display ±1 SEM.

2-(8-bromo-2.3,6,7-tetrahydrobenzo [1,2-b:4,5-b′]difuran-4-yl)-*N-*[(2-methoxybenzyl)ethan-1-amine (2C-B-Fly-NBOMe, Figure 1) represents a newly arising compound from the NBOMe series. It was first mentioned in a poster describing the development of new and highly potent 5-HT_2A_ receptor agonists and further researched in a dissertation by Ralf Heim (Elz et al., 2002; Heim, 2003). In a positron emission tomography study in Danish Landrace pigs, 2C-B-Fly-NBOMe readily penetrated the brain tissue; it had the highest cortical uptake among the examined NBOMes. Parallel *in vitro* competition binding study in GF-62 cells showed the highest binding affinity of 2C-B-Fly-NBOMe to 5-HT_2A_ receptor, although its intrinsic activity was surprisingly the lowest (Ettrup et al., 2011). *In vitro* analysis of its metabolites by Richter et al. (2020) found five phase I metabolites and one phase II metabolite in human hepatocellular carcinoma cells. A recent study from our team describes thirty-five phase I and nine phase II metabolites in three different systems–isolated human liver microsomes, *Cunninghamella elegans* mycelium and Wistar rats (Nykodemová et al., 2021).

Despite two decades have passed, there is still a critical deficit of scientific data concerning this analogue, and to the best of our knowledge, no study has focused on the changes in behavioural and physiological parameters after 2C-B-Fly-NBOMe intake up until now. So far, it is a non-controlled substance in most countries, yet data from online drug forums already suggest apparent interest in 2C-B-Fly-NBOMe, even though consequences of such ingestion are unknown and could be life-threatening, given its presumed high potency.

The aim of this study was thus to determine the pharmacokinetic profile of 2C-B-Fly-NBOMe in serum and brain over a 24 h period, and acute effects on locomotor behaviour in the open field test (OFT), sensorimotor gating in the prepulse inhibition (PPI) of acoustic startle response (ASR) and body temperature in adult male Wistar rats. As this research was part of a larger battery of experiments with NPS, we consequently used a comparable design of two temporal onsets for the behavioural experiments—15 and 60 min after the drug administration. This range is favourable for detecting a wide spectrum of acute behavioural effects. Based on our previous studies with NPS and classical serotonergic psychedelics (Páleníček et al., 2008; Páleníček et al., 2010; Páleníček et al., 2013; Tylš et al., 2016; Šíchová et al., 2022) and the literature evidence from other NBOMe analogues (Gatch et al., 2017; Halberstadt, 2017; Halberstadt and Geyer, 2017; Miliano et al., 2019), we expect that 2C-B-Fly-NBOMe is able to induce an inhibitory or biphasic effect on locomotion, and a deficit in sensorimotor gating with effects being more pronounced at 60 min after administration due to reported gradual onset of action of NBOMe substances. According to a series of case reports describing NBOMe intoxications with signs of serotonin toxicity, we also hypothesize that 2C-B-Fly-NBOMe induces hyperthermia, especially in group-housed animals–a laboratory setting mimicking crowded dance club conditions, allowing to explore possible aggregation toxicity of the investigated drug (Fantegrossi et al., 2003; Saadat et al., 2005).

## 2 Materials and methods

### 2.1 Animals

All experiments were carried out on adult male Wistar rats (Velaz, Prague, Czechia). Animals were housed in pairs in controlled conditions (12/12 h light/dark regime with light phase from 6:00 h to 18:00 h, temperature 22°C ± 2°C, humidity 30%–70%) with access to standard diet and water *ad libitum*. Rats were given an acclimatization period of 1 week prior to the start of each experiment, during which they were weighed twice and handled four times; the rats were approximately 9 weeks old and weighed approx. 200–250 g at the time of testing. Each experimental group consisted of 10 rats based on our previous studies with a standardized protocol for comparable data (Rohanová et al., 2008; Páleníček et al., 2013; Šíchová et al., 2022), and each subject was tested only once according to the principle of acute behavioural testing, except for the pharmacokinetic studies–to minimize the number of sacrificed animals, rats from behavioural experiments treated with saline and 1 mg/kg of 2C-B-Fly-NBOMe were used for the determination of drug concentrations in brain and serum. Experiments were conducted during the light phase of the cycle; animals were brought to the testing rooms with a controlled temperature (22°C ± 2°C) 1 h before the start of each experiment for acclimatization. All experiments respected the Guidelines of the European Union (86/609/EU) and the protocol was approved by the National Committee for the Care and Use of Laboratory Animals (Czechia) under no. 59449/2016-MZE-17214.

### 2.2 Drugs and chemicals

2C-B-Fly-NBOMe was synthesized, purified, and subsequently converted to 2C-B-Fly-NBOMe hydrochloride by the University of Chemistry and Technology in Prague at the Forensic Laboratory of Biologically Active Substances. The resulting hydrochloride was certified to be of 99% purity (analysed by high-performance liquid chromatography) and served as a reference standard for pharmacokinetic analyses using liquid chromatography/mass spectrometry (LC/MS). Chemicals used in a LC/MS analysis were of LC/MS grade and together with other necessary chemicals purchased from Merck (Czechia). Ultrapure water, 18.2 MΩ/cm, was produced by the Smart2Pure 12 UV system (Thermo Scientific, Germany). Homogenization beads, SiLibeads, type ZY 0.4–0.6 mm, were supplied by Ginzel (Czechia). Stock solutions of analytical standards were prepared in methanol and stored at −20°C. 2C-B-Fly-NBOMe was stored in a dry, dark place and then dissolved in deionized water and 20 μl of Tween 20 just before the experiments. The highest dose was briefly dissolved using an ultrasonic homogenizer due to poor solubility.

### 2.3 Dosage

Based on previously published behavioural studies with 25B-NBOMe, 25C-NBOMe and 25I-NBOMe (Halberstadt and Geyer, 2014; Elmore et al., 2018; Nakamura et al., 2018; Miliano et al., 2019) our experience with related compound 25CN-NBOMe (Šíchová et al., 2022), and reports on selectivity and potency of 2C-B-Fly-NBOMe on 5-HT_2A_ receptors, a range of three concentrations was estimated for the experiments: 0.2, 1 and 5 mg/kg s. c. dissolved and administered in a total volume of 2 ml/kg. An equivalent volume (2 ml/kg) of deionized water and 20 μl of Tween 20 was used as negative control (ctrl).

### 2.4 Pharmacokinetic analysis

For the pharmacokinetic study, rats were treated with 1 mg/kg s. c. of 2C-B-Fly-NBOMe and then sacrificed by decapitation after 0.5, 1, 2, 4, 8 and 24 h (n = 8 per time point). Collected blood was left to clot in a refrigerator for 1 h, then centrifuged for 10 min at 1500 g, 10°C, and the separated serum was collected. Whole brains were excised manually using surgical instruments. All serum and brain samples were stored at −20°C until the analysis.

#### 2.4.1 Determination of 2C-B-fly-NBOMe levels in serum and brain samples using LC/MS

##### Serum pre-treatment

200 μl of blood serum was diluted in a microtube with 200 μl of 1% ammonia solution and homogenized in a Bullet Blender tissue homogenizer (speed 2, time 5 min). Next, 400 μl of ethyl acetate was added by the automatic dispenser and the whole content was homogenized again (speed 3, time 5 min), additional 400 μl of ethyl acetate was added and the sample was homogenized for the last time. The homogenate was centrifuged (10 min, 25°C, 12,000 RPM), 600 μl of the top layer was transferred into a new microtube and 50 μl of 10% formic acid solution was added. After proper shaking, the solution was evaporated in the CentriVap concentrator. The residue was then dissolved in 100 μl of methanol and shaken for 10 min. Prior to the analysis, the solution was diluted 1:1 in deionized water, mixed and centrifuged.

##### Tissue pre-treatment

100 µL of 1% ammonium hydroxide solution was added to 200 mg of brain tissue. A mixture was homogenized with 200 mg of zirconium oxide beads in a BulletBlender for 10 min (speed 5). 400 μL of ethyl acetate was added to the mixture and homogenized (BulletBlender, speed 3, time 5 min). Additional 400 µL of ethyl acetate was added to the mixture and homogenized (BulletBlender, speed 3, time 5 min). Samples were centrifuged (10 min, 25°C, 12,000 RPM), 400 µL of supernatant was acidified and completely evaporated (Centrivap Concentrator). The dry residue was reconstituted with 400 µL of methanol. Prior to the analysis, 250 µL of the solution was diluted with 750 µL of water, mixed, and centrifuged (0.2 µm PVDF filter).

##### LC/MS conditions

The samples were analysed using ultra-high performance liquid chromatography-tandem mass spectrometry instrumentation (1,290 Infinity—6,460 Triple Quadrupole LC/MS Agilent Technologies with AJS electrospray ionization source). A column Kinetex Biphenyl (100 × 2.1 mm, 2.6 µm) with a pre-column was used for a chromatographic separation at 35°C with gradient elution in a system of 5 mM ammonium formate with 0.01% (v/v) formic acid (mobile phase A) and methanol with 0.01% (v/v) formic acid (mobile phase B). The gradient started at 10% B, increased from 10% to 50% B in 0.5 min and from 50% to 100% B in 1.5 min, held for 2 min at 100% B, decreased to 10% B in 0.5 min and held for 2 min at 10% B. Data were acquired in positive electrospray ionization (ESI) mode using a multiple reaction monitoring method, one quantification ion and three confirmation ions were used. 2C-B-Fly-NBOMe was quantified using an external matrix-matched calibration. The limit of quantification was 1 ng/ml (1 ng/g for brain tissue). The matrix-matched calibration was performed in duplicates; real samples were also processed in duplicates.

### 2.5 Behavioural experiments

#### 2.5.1 Open field test

OFT was performed in a black square plastic arena (80 × 80 × 40 cm) in an evenly lit and sound-proof testing room. Rats were individually placed into the centre and allowed to explore the arena for 30 min while their behaviour was recorded. The experimental arena was cleaned with 70% ethanol after each subject. Arena was virtually divided into a 5 × 5 grid of identical squares; 16 squares were located next to the walls (peripheral zone) and 9 squares were in the centre (central zone). Software EthoVision XT 14.0 software (Noldus, Netherlands) was used to capture raw data used in the calculation of the following dependent variables: trajectory length (cm; corrected for deviations of <3 cm) and its temporal dynamics in 5 min intervals; thigmotaxis (∑f_peripheral zones_/∑f_all zones_, f = frequency of appearance in the zone) reflecting the probability of appearance in the peripheral zone; T_center_ reflecting time spent in the centre of the arena (∑time_central zones_).

#### 2.5.2 Prepulse inhibition of acoustic startle response

The effect of 2C-B-Fly-NBOMe on sensorimotor gating was evaluated by a PPI of ASR test. Two identical startle chambers (SR-LAB, San Diego Instruments, California) were used, each containing a sound-proof, evenly lit and ventilated enclosure with a plexiglass stabilimeter (8.7 cm inner diameter), a piezoelectric accelerometer for detecting amplitudes of the ASR, and a high-frequency loudspeaker producing acoustic stimuli. Amplitudes of the ASR were digitized for subsequent analysis. The experimental design was based on our previous studies (Štefková et al., 2017; Šíchová et al., 2018). Two days before the experiment, the animals were habituated to the startle chambers with a background white noise (75 dB) for 5 min, followed by six presentations of pulse-alone stimuli (125 dB, 40 ms) with pseudorandomized inter-stimulus intervals. The test itself comprised a habituation period (5 min, 75 dB), followed by six pulse-alone stimuli (125 dB, 40 ms) for establishing baseline ASR; then 60 trials delivered in pseudorandomized order: (A) pulse alone: 125 dB, 40 ms; (B) prepulse-pulse: 83 or 91 dB; 20 ms prepulse; variable (30, 60, or 120 ms) inter-stimulus interval (ISI) (mean 70 ms), followed by 125 dB, 40 ms pulse; and (C) no stimulus. Finally, six pulse-alone trials (125 dB, 40 ms) were delivered.

Habituation was calculated by the percentage reduction in the ASR from the initial six baseline trials to the final six trials. PPI was calculated as [100 - (mean response for the prepulse - pulse trial/startle response for the single pulse trials) × 100].

Animals with mean ASR response lower than 10 arbitrary units were excluded from the analysis as non-responders.

### 2.6 Thermoregulation

For the assessment of rectal temperature changes, rats were housed individually or in groups of five per cage. They were treated with 2C-B-Fly-NBOMe (5 mg/kg) or ctrl solution s. c. at 9:00 h. During the measurements, each rat was briefly immobilized in a plexiglass tube and then returned to its home cage. In total, 14 measurements were performed: 3 hourly measurements (07:00–09:00 h) for setting the baseline temperature, and then 4 subsequent 30 min measurements (09:30–11:00 h) and 6 hourly measurements (12:00–17:00 h).

### 2.7 Design and statistical analysis

Statistical analyses were performed in STATISTICA (version 13.3, StatSoft, Inc.), R software (version 4.0.5) and R Studio (version 1.4.1717). Tests used a default alpha set at *p* = 0.05, two-tailed.

For the pharmacokinetic data, a one-compartment model with a first-order absorption equation (Wijnand, 1988) was fitted using a cubic spline interpolation:

$$C\left(t\right)=D\cdot ka\cdot \frac{\frac{F}{V}}{ka-ke}\times \left[\exp\left(ke\cdot t\right)-\exp(-ka\cdot t\right]$$

Where *C* = 2C-B-Fly-NBOMe concentration, *t* = time, *D* = dose, *ka* = absorption rate constant, *F* = bioavailability, *V* = volume of distribution, *ke* = elimination rate constant.

The half-life of the drug elimination was calculated using Package PKNCA version 0.9.4 implemented in R software (Buckeridge et al., 2015).

Behavioural and thermoregulatory data were analysed using analysis of variance (ANOVA) with a factorial design. Significant main effects and inter-factor interactions were followed by pairwise comparison using Tukey *post hoc* test.

Locomotor activity in OFT (mean trajectory length across 5 min blocks) was evaluated using a 2 × 4  ×  6 mixed factorial ANOVA with temporal testing onset (15 or 60 min) and drug treatment (2C-B-Fly-NBOMe 0.2, 1, or 5 mg/kg or ctrl) as between-subjects factors, and time blocks (6 × 5 min intervals) as a within-subjects factor. Spatial distribution of animal movement in OFT (thigmotaxis and *T*_center_) and PPI parameters (habituation, ASR and % of PPI) were each analysed using a 2 × 4 factorial ANOVA with temporal testing onset (15 or 60 min) and drug treatment (2C-B-Fly-NBOMe 0.2, 1, or 5 mg/kg or ctrl) as between-subjects factors.

Data from thermoregulatory experiments were analysed using 2 × 2 × 14 mixed factorial design with drug treatment (2C-B-Fly-NBOMe at 5 mg/kg or ctrl) and home cage conditions (individually and group-housed animals) as between-subjects factors and time (14 measurements) as a within-subjects factor. The baseline temperatures of animals were compared in the same manner.

## 3 Results

### 3.1 Pharmacokinetics

Maximum mean 2C-B-Fly-NBOMe concentration was attained within 0.5 in the blood serum (28 ng/ml) and 1 h in the brain (171 ng/g) with a half-life of elimination of 1.56 h and 2.40 h, respectively. The drug was almost undetectable in the serum after 8 h, while it was still present in the brain tissue (21 ng/g; Figure 1A). More pharmacological parameters are shown in Table 1.

**TABLE 1:** Overview of pharmacokinetic parameters after 1 mg/kg s. c. 2C-B-Fly-NBOMe application. Data are presented as mean ± SEM.

Parameters	Serum	Brain	Units
C_max_	28.44 ± 6.33	159.15 ± 15.34	ng/ml; ng/g
T_max_	0.8	1.1	h
t_1/2_	2.55	3.2	h
AUC0-24 h	115.3 ± 25.6	826.3 ± 112.6	h·ng/ml; h·ng/g
F (%)	0.961 ± 0.048	0.698 ± 0.035	%
V d (mL)	7.231 ± 0.362	0.559 ± 0.028	mL
ka (h^-1^)	0.731 ± 0.036	0.685 ± 0.034	h^-1^
ke (h^-1^)	0.733 ± 0.037	0.655 ± 0.033	h^-1^

Abbreviations: F, bioavailability; Vd, volume of distribution; ka, absorption rate constant; ke, elimination rate constant.

### 3.2 Open field test

Analysis of total trajectory length revealed a main effect of drug treatment [F (3.72) = 64.91, *p* < 0.001], temporal onset [F (1,72) = 13.42, *p* < 0.001], as well as their interaction [F (3,72) = 4.55, *p* < 0.01]. *Post hoc* tests revealed significant differences in all remaining groups *versus* ctrl except for 0.2 mg/kg of 2C-B-Fly-NBOMe in the 60 min onset.

Further analysis of locomotion in 5 min time blocks displayed a significant main effect of time blocks [F (5,360) = 86.72, *p* < 0.001], and interactions of time blocks × treatment [F (15,360) = 16.01, *p* < 0.001], however triple interaction, treatment × testing onset × time blocks was not significant. Locomotor habituation was dose-dependently disturbed in all 2C-B-Fly-NBOMe groups in both testing onsets. Compared to ctrl, at the 15 min temporal onset, all the doses significantly decreased activity in the first three blocks (*p* < 0.001). In the 1 mg/kg group, the effect persisted until the fourth block (*p* < 0.05). The highest dose of 5 mg/kg caused significant hypolocomotion during the whole session (*p* < 0.05–0.001) (Figure 1C). At 60 min testing onset, all groups treated with 2C-B-Fly-NBOMe displayed reduced activity in the first two blocks (*p* < 0.05–0.001), which persisted to the fifth block in 1 mg/kg and 5 mg/kg groups (*p* < 0.01–0.001, Figure 1D). No significant differences were observed between the groups in the sixth block. For a graph of total locomotion and visualisation of trajectory patterns, see Figure 1B. Both thigmotaxis and T_centre_ were not affected either by treatment, testing onset or their interaction.

### 3.3 PPI of ASR

Three animals from the 5 mg/kg group in 15 min onset and two animals from 5 mg/kg in 60 min onset were excluded from the analysis because of low mean ASR values. Habituation was significantly affected by drug treatment [F (3,67) = 2.79, *p* < 0.05], but not by temporal onset or by the interaction of these factors. ASR was significantly affected by drug treatment [F (3,67) = 25.11, *p* < 0.001], but not by testing onset or interaction of these two factors. *Post hoc* test revealed that all doses of 2C-B-Fly-NBOMe significantly diminished ASR in comparison to ctrl (*p* < 0.001; Figure 1E). The percentage of PPI was significantly affected by testing onset [F (1, 67) = 6.65, *p* < 0.05], but not by treatment or by temporal onset × treatment interaction (Figure 1F).

### 3.4 Body temperature

Baseline body temperatures did not significantly differ between the groups. A significant effect of the treatment on body temperature was observed [F (1,36) = 7.00, *p* < 0.05]. Furthermore, the home-cage condition factor was significant [F (1,36) = 14.79, *p* < 0.001]. The interaction of time x home-cage condition was also significant [F (13,468) = 1.98, *p* < 0.05], triple interaction of time x home-cage condition x time of measurement was not significant. Despite the hypothermic trend in individually housed animals during the first 90 min after s. c. application (10:00–10:30, Figure 1G), the statistical difference was not significant.

## 4 Discussion

2C-B-Fly-NBOMe rapidly entered the blood system; peak concentrations were detected 30 min and 1 h after the application in serum and brain, respectively. The drug was markedly present in the brain tissue for up to 8 h 2C-B-Fly-NBOMe induced strong and dose-dependent decrease of locomotor activity and marked disruption of ASR throughout the testing window (at 15 as well as 60 min testing onsets), but also a disruption of PPI in 15 min testing onset. In contrast, the animals did not demonstrate any behaviour generally acknowledged as a typical phenotype connected to 5-HT2A receptor activation by psychedelics (head-twitch response or wet dog shakes).

### 4.1 Pharmacokinetics

In our previous study, parental drug 2C-B (50 mg/kg s. c.) showed a similar pharmacokinetic profile, with peak concentrations attained within 30 min in blood serum and 60 min in brain, liver, and lung tissue. Presence of 2C-B was detectable for up to 5 h in all collected tissues (Rohanová et al., 2008). A recent investigation of two novel “FLY” drugs (1 mg/kg, peroral administration) by Baralla et al. (2019) showed their peak concentrations in 30 and 60 min for 1-(2,3,6,7-tetra-hydrofuro [2,3-*f*][1]benzofuran-4-yl)propan-2-amine (FLY) and 2-(2,3,6,7-tetrahydrofuro [2,3-*f*][1]benzofuran-4-yl)ethanamine (2C-FLY), respectively, in rat blood serum, with expressly distinct peak values. Our results show a markedly higher concentration of 2C-B-Fly-NBOMe in the brain tissue compared to the blood serum, suggesting that 2C-B-Fly-NBOMe readily passes through the blood-brain barrier. The long presence of 2C-B-Fly-NBOMe in the brain tissue is supportive of the prolonged behavioural and physiological effects of NBOMes in humans. Indeed, self-reports of recreational users declare peak effects of various NBOMes within 45–90 min and duration for up to 8 h (Lawn et al., 2014). Our recent study of closely related substance 25CN-NBOMe determined half-life values of 1.88 h for the blood serum and 2.28 h for the brain tissue, however, in a five-fold higher applied dose of 5 mg/kg (Šíchová et al., 2022). A higher half-life value of 2C-B-Fly-NBOMe in the brain might indicate its slower turnover and possibly a longer mechanism of action of the drug. Explanation of the concentration peak after 1 h in the brain tissue compared to the strong behavioural effects observed already 15 min after the drug administration may lay in a formation of potentially active metabolites (Stellpflug et al., 2014), after all, recent studies focused on 2C-B-Fly-NBOMe observed massive process of 2C-B-Fly-NBOMe’s metabolization (Richter et al., 2020; Nykodemová et al., 2021).

### 4.2 Locomotor activity

Several studies observed a biphasic effect of psychedelics such as LSD (Adams and Geyer, 1982), 2C-B (Páleníček et al., 2013) and mescaline (Páleníček et al., 2008). Generally, this effect can be described as the drug’s ability to act differently in low and high doses, as well as in different temporal onsets. These mechanisms presumably involve differential 5-HT_2A/2C_ receptor activation and subsequent alteration of dopaminergic neurotransmission, as dopamine is the key neurotransmitter responsible for motor control (Páleníček et al., 2013). 5-HT_2A_ receptors are most likely involved in the onset of hyperlocomotion (Ninan and Kulkarni, 1998; Halberstadt et al., 2013), whereas 5-HT_2C_ receptors possibly mediate the inhibitory effect on locomotion (Bagdy et al., 2001; Harvey-Lewis et al., 2016). In a recent study, 25I-NBOMe induced a biphasic effect after s. c. application, but interestingly not after i. p. injection (Halberstadt, 2017). The dose of 0.1 mg/kg s. c. induced significant hyperlocomotion in 60 min temporal onset, whereas 3 mg/kg s. c. caused hypolocomotion in mice. Other examinations imply that NBOMe substances, namely 25B-NBOMe, 25C-NBOMe and 25I-NBOMe, when administered i. p., cause a dose-dependent decrease in locomotor activity (Gatch et al., 2017; Wojtas et al., 2021), which is in correlation to our observation. 25CN-NBOMe manifested a similar, yet less pronounced decrease in locomotion (Šíchová et al., 2022). It is of note that the inhibitory effect of 2C-B-Fly-NBOMe was so prominent that the animals were unable to exhibit typical thigmotactic behaviour, especially in higher doses.

### 4.3 PPI of ASR

Activation of 5-HT_2A_ receptors leads to a disruption of sensorimotor gating (Sipes and Geyer, 1997), and serotonergic 5-HT_1A_ and 5-HT_2C_ receptors may also be of importance in this mechanism (Krebs-Thomson et al., 2006). Interestingly, a biphasic effect on PPI can be observed, depending on the dose or the temporal onset (Davis and Walters, 1977; Páleníček et al., 2013). Examination of 25I-NBOMe by Miliano et al. (2019) revealed a significant decrease of ASR in the highest dose (1 mg/kg i. p.) and dose-dependent inhibition of PPI in rats. Similarly, 25CN-NBOH also showed tendencies to disrupt PPI in rats (Halberstadt and Geyer, 2017). 25CN-NBOMe affected PPI only in the highest dose of 5 mg/kg in the 15 min onset (Šíchová et al., 2022). According to our data, 2C-B-Fly-NBOMe strongly attenuates ASR even in the lowest dose of 0.2 mg/kg, which is fully in line with our previous observations with 2C-B. Interestingly, the effect on PPI was not very prominent–rather than showing any significant effect of the drug, the results might stem from the different temporal onsets and animals’ reactions to the irritating environment.

### 4.4 Thermoregulatory effect

Generally, 5-HT_2A_ receptor agonists cause elevations in body temperature, which can lead to severe hyperthermia as one of the main symptoms of serotonin syndrome (Menon and Dandiya, 1969; Murakami and Sakata, 1980; Gudelsky et al., 1986; Matuszewich and Yamamoto, 2003). According to available data from case studies of severe intoxications, NBOMe substances indeed induce hyperthermia, occasionally up to the point where active cooling of the patient’s body must be performed (Andreasen et al., 2015; Gee et al., 2016). This effect is possibly caused by their agonism on α-adrenergic receptors, leading to vasoconstriction (Tang et al., 2014). A study of 25B-NBOMe [(0.25 intraperitoneally (i. p.)] in rats suggested direct involvement of peripheral 5-HT_2A_ receptors in hyperthermia, but only in higher ambient temperature (29°C; Nakamura et al., 2018). In another study made by Miliano et al. (2019), a medium dose of 25I-NBOMe (0.5 mg/kg i. p.) induced hyperthermia within 35–65 min in female rats, but not in males. In contrast, we observed a trend of hypothermic reaction in individually housed animals, but not in animals housed in groups. A significant hypothermic effect was observed after 25CN-NBOMe application (5 mg/kg) in individually-housed animals (Šíchová et al., 2022). Thus, there might be a different molecular mechanism of action connected to other receptors’ agonism. It is known that 5-HT_1A_ receptors cause a decrease in body temperature (Papeschi et al., 1971; Gudelsky et al., 1986) however, according to the binding study with similar NBOMe compounds by Rickli et al. (2015), affinity to this receptor subtype is considerably diminished. Another explanation may be in the modulation of different receptors involved in thermoregulatory processes. An extreme attenuation of the movement caused by the drug could be an important factor in these results, possibly in combination with circadian rhythms, as rats’ body temperature tends to decline during the daytime (Kittrell and Satinoff, 1986). The question remains whether the results were influenced by the stress induced by repeated measurements of the animals’ temperature, although other studies from our group using the same method had shown demonstrable effects of drugs on body temperature (Páleníček et al., 2011; Štefková et al., 2017; Šíchová et al., 2022).

In conclusion, 2C-B-Fly-NBOMe is a substance with a very similar profile to 25CN-NBOMe, and to some extent other drugs from the NBOMe family, when compared with other preclinical studies. As expected, the drug readily crossed the blood-brain barrier and persisted in the system for several hours, similar to human reports. In contrast to the reports of recreational users with NBOMe compounds usually enhancing physical activity almost to the state of agitation, 2C-B-Fly-NBOMe caused strong inhibitory effects in our study. Thermoregulation also appears to be affected differently in rats compared to humans, suggesting different patterns of action in different species or a significant role of environmental factors.

## Data Availability

The raw data supporting the conclusions of this article will be made available by the authors, without undue reservation.
